# Testing the habituation assumption underlying models of parasitoid foraging behavior

**DOI:** 10.7717/peerj.3097

**Published:** 2017-03-16

**Authors:** Paul K. Abram, Antonino Cusumano, Katrina Abram, Stefano Colazza, Ezio Peri

**Affiliations:** 1 Université de Montréal, Institut de Recherche en Biologie Végétale, Montréal, Canada; 2 Dipartimento di Scienze Agrarie e Forestali, Università degli Studi di Palermo, Palermo, Italy; 3 Department of Entomology, Wageningen University, Wageningen, The Netherlands

**Keywords:** Learning, Optimal foraging theory, Habituation, Patch exploitation, Infochemical cues

## Abstract

**Background:**

Habituation, a form of non-associative learning, has several well-defined characteristics that apply to a wide range of physiological and behavioral responses in many organisms. In classic patch time allocation models, habituation is considered to be a major mechanistic component of parasitoid behavioral strategies. However, parasitoid behavioral responses to host cues have not previously been tested for the known, specific characteristics of habituation.

**Methods:**

In the laboratory, we tested whether the foraging behavior of the egg parasitoid *Trissolcus basalis* shows specific characteristics of habituation in response to consecutive encounters with patches of host (*Nezara viridula*) chemical contact cues (footprints), in particular: (i) a training interval-dependent decline in response intensity, and (ii) a training interval-dependent recovery of the response.

**Results:**

As would be expected of a habituated response, wasps trained at higher frequencies decreased their behavioral response to host footprints more quickly and to a greater degree than those trained at low frequencies, and subsequently showed a more rapid, although partial, recovery of their behavioral response to host footprints. This putative habituation learning could not be blocked by cold anesthesia, ingestion of an ATPase inhibitor, or ingestion of a protein synthesis inhibitor.

**Discussion:**

Our study provides support for the assumption that diminishing responses of parasitoids to chemical indicators of host presence constitutes habituation as opposed to sensory fatigue, and provides a preliminary basis for exploring the underlying mechanisms.

## Introduction

As animals experience repeated environmental stimuli that become irrelevant or unreliable, they often show less intense behavioral responses as a result (Thompson & Spencer, 1966; Pinsker et al., 1970; Carew, Pinsker & Kandel, 1972). This form of non-associative learning, termed “habituation”, is ubiquitous among animals. Although habituation is often referred to as a “simple” form of learning, this view is probably unjustified in light of the clear complexity and diversity of the neurological and molecular mechanisms involved (Rankin et al., 2009). Furthermore, habituation has been shown to be involved in ecologically important behavioral responses to factors including predation risk (Deecke, Slater & Ford, 2002) and conspecific acoustic signaling (Owen & Perrill, 1998; Dong & Clayton, 2009).

Habituation shows a well-defined set of characteristics that apply to many different physiological and behavioral responses (reviewed by Thompson & Spencer, 1966; Rankin et al., 2009). These characteristics include decreasing response intensity following repeated experiences with a given cue, and a “spontaneous recovery” of the response over time after stimulation ceases. Furthermore, both the decline and the recovery of habituated responses tend to be more rapid and more pronounced when habituation training takes place at shorter intervals. These defining characteristics are useful for distinguishing habituation from sensory fatigue—wherein sensory structures become saturated by chemical, visual, or acoustic stimuli (and do not result in training interval-dependent decline and recovery) as well as motor fatigue (Rankin et al., 2009). These specific characteristics of habituation have been the subject of considerable study by comparative physiologists investigating relatively simple behaviors (e.g., startle responses, escape reactions, gill withdrawal, proboscis extension) over short time periods (Byrne, 1982; Braun & Bicker, 1992; Rankin & Broster, 1992; Engel & Wu, 2009), but have been neglected by behavioral ecologists studying more complex behaviors such as foraging.

Parasitoid wasps, whose offspring develop on or inside other insects and kill them as a result (Quicke, 1997), are important model organisms in behavioral ecology—particularly for testing hypotheses related to optimal foraging theory (Wajnberg, 2006). These insects have evolved sophisticated behavioral strategies to locate their hosts in complex environments, many of which involve learning. Learning is particularly important for foraging parasitoids in the context of detection of infochemical cues (e.g., synomones, kairomones) that can serve as potential indicators of host presence (Vet & Dicke, 1992; Fatouros et al., 2008; Colazza et al., 2014; Giunti et al., 2015). While it may initially be advantageous for a parasitoid to allocate foraging time to a patch or habitat containing infochemical cues from its host, parasitoids should eventually leave if hosts cannot be found or if a previously profitable patch becomes depleted. For example, in the classic mechanistic model for patch time allocation developed by Waage (1979), parasitoids enter a host patch with an initial level of responsiveness to host-derived contact kairomones (whose concentration is proportional to host density). The parasitoid’s responsiveness, which determines its motivation to remain in the host patch, declines progressively over time but can be temporarily increased when the female parasitoid lays an egg in or on a host, updating her estimation of patch quality. Waage (1979) suggested habituation as the mechanism underlying parasitoids’ decreasing responsiveness to host kairomones, and this hypothesis has since been echoed many times in both empirical tests and theoretical refinements of the original model (e.g., Bouchard & Cloutier, 1984; Strand & Vinson, 1982; Takabayashi & Takahashi, 1989; Vet & Dicke, 1992; Pierre, Van Baaren & Boivin, 2003; Louâpre & Pierre, 2014). Despite the fact that this assumption has become the conventional wisdom in parasitoid foraging theory (Vet & Dicke, 1992; Van Alphen, Bernstein & Driessen, 2003; Wajnberg, 2006) no studies have tested whether parasitoid behavioral responses to contact kairomones actually show the defined characteristics of habituation (as opposed to sensory fatigue, for example). Thus, the “habituation assumption” represents a major untested mechanistic component of important optimal foraging theory models.

The mechanisms underlying learning and memory are often explored by experimentally blocking neurological processes leading to memory consolidation. These experiments can be used to probe the different phases involved in various forms of memory (Margulies, Tully & Dubnau, 2005), and their potential stability in the face of environmental stressors (Teskey et al., 2012; Abram et al., 2015). In parasitoid wasps as well as other animals, most of this work has focused on memory resulting from associative learning (e.g., Hoedjes et al., 2011; Teskey et al., 2012). Associative learning-derived memory in insects is characterized by several, often overlapping, phases including short-term memory (STM), anesthesia-resistant memory (ARM), and long-term memory (LTM) (Margulies, Tully & Dubnau, 2005; Van den Berg et al., 2011; Hoedjes et al., 2011). Cold anesthesia, ATPase inactivators, and protein synthesis inhibitors, respectively, have been shown to disrupt these three memory phases and cause amnesia (Xia, Feng & Guo, 1998; Xia, Feng & Guo, 1999; Smid et al., 2007; Van den Berg et al., 2011; Kruidhof et al., 2012; Hoedjes & Smid, 2014; Smid & Vet, 2016). Although associative learning and habituation may have similar underlying mechanisms (e.g., Duerr & Quinn, 1982; Engel & Wu, 1996; Cho, Heberlein & Wolf, 2004; Asztalos, Arora & Tully, 2007), it is unclear whether habituation-derived memory in insects might also show phases of consolidation that are sensitive to disruption by similar means.

In this study we tested for the characteristics of habituation in parasitoid foraging behavior, using *Trissolcus basalis* (Hymenoptera: platygastridae) as a model organism. *Trissolcus basalis* is a minute (∼1 mm in body length) parasitoid of stink bug eggs distributed throughout several regions of the world. Its mostly closely associated host, *Nezara viridula* (Hemiptera: Pentatomidae), is a polyphagous pest of a variety of crop plants, notably soybean (Todd, 1989). *Trissolcus basalis* utilizes a range of indirect infochemical cues to locate host egg masses, including footprints (cuticular hydrocarbons) deposited by hosts on plant substrates while walking (Colazza et al., 2014). Detection of host footprints by *T. basalis* induces a behavioral arrestment response (slower walking and increased turning tendency) that has been extensively studied in this species (e.g., Peri et al., 2006; Abram et al., 2015). As in the Waage (1979) model, the arrestment response of *T. basalis* decreases in intensity after an unrewarded experience with host footprints, but can be restored by oviposition (Peri et al., 2006). The behavioral expression of the memory of an unrewarded experience (i.e., decreased residence time, increased walking speed, decreased turning tendency) persists for about 72 h, although it can be extended by exposure to stressful temperatures (Peri et al., 2006; Abram et al., 2015). We assessed whether *T. basalis’* behavioral response to host footprints demonstrates the following characteristics of habituation: (i) the arrestment response of *T. basalis* progressively declines over a series of several consecutive training sessions on host footprints; (ii) the decline in responsiveness is greater after shorter training intervals; (iii) the arrestment response fully or partially recovers (i.e., spontaneous recovery) after different intervals following the training sessions; (iv) the rapidity and degree of spontaneous recovery is higher following shorter-interval training. As a first step towards unraveling the mechanisms underlying this putative habituation learning and its sensitivity to disruption compared to previously studied forms of parasitoid learning (i.e., associative learning), we also tested whether several treatments (cold anesthesia, ATPase inhibition, protein synthesis inhibition) that have been used to disrupt invertebrate memory in past studies could disrupt this putative habituation-derived memory. Our study represents the first explicit test for specific characteristics of habituation in a parasitoid’s behavioral responses to host cues.

## Materials and Methods

### Insect rearing

*Nezara viridula* and *T. basalis* were reared as described by Abram et al. (2015). The day before female *T. basalis* were used in experiments, they were isolated from the colony and placed in a 0.2 mL vial with a drop of pure liquid honey for food and water. Females had no oviposition experience, had been mated, and were 2–6 days old when used in trials. At this age, females are ready to parasitize hosts but are still in the early stages of their adults lives (*T. basalis* lives up to several months in the laboratory). Insect colonies were maintained at 26 ± 1 °C, 16:8 h L:D, and 60 ± 10% RH.

### General bioassay procedure

To expose *T. basalis* to host footprints and measure their behavioral response in all of the experiments, we used methods described in detail elsewhere (Peri et al., 2006; Abram et al., 2015). Briefly, tests were conducted between 0900 and 1300 h in an isolated room (temperature: 25 ± 1 °C) with standardized lighting, in open arenas consisting of a 25 × 25 cm sheet of filter paper. A circular area (diameter: 6 cm) in the middle of the arena was exposed to a single mated female *N. viridula* for 30 min, in order to treat it with the stink bug’s footprints (the rest of the arena was left untreated). *Trissolcus basalis* females were released individually into the centre of the treated arena by opening and gently tapping the vial, which immediately induced the arrestment response when the wasp contacted the filter paper. Wasps were observed until they walked off of the filter paper. The residence time (the time between release and leaving the arena) of each wasp was recorded, and filter paper was renewed. Residence time has previously been shown to be a reliable indicator of the intensity of parasitoid arrestment responses (longer residence times indicating a more intense arrestment response), being strongly negatively correlated with average walking speed and positively correlated with turning tendency (e.g., Peri et al., 2006).

### Tests for characteristics of habituation

First, we characterized the decline in the intensity of the parasitoid’s arrestment response (i.e., residence time) over a series of consecutive exposures to patches of host footprints (Characteristics I–II in last paragraph of introduction). Wasps were trained on four subsequent patches of host footprints (released into an arena, allowed to leave, and re-collected) with either 15 min, 30 min, or 60 min between training sessions (hereafter, “training interval”). Wasps were held in vials with a drop of liquid honey between training sessions, and following the conclusion of the entire series of four training sessions.

To measure the recovery of their arrestment response after training and its dependence on training interval (Characteristics III–IV), wasps were randomly assigned to two groups, and re-tested either 24 h or 48 h (during which they were held in vials with liquid honey) after the conclusion of their final training session (hereafter, “testing interval”). In parallel, as a control for training experience, we measured the residence times of untrained individuals that were isolated from the colony at the same time as the trained individuals and assayed at the same testing interval, but had not previously been exposed to host footprints. We have previously verified that the experience of being released into the arena (versus being confined to the tube), independent of exposure to host footprints, does not affect the subsequent responses of wasps (unpublished data). In total, we obtained training data for 160 (40 per training interval) wasps, and testing data for a total of 212 wasps (92 trained, 120 untrained).

### Memory disruption tests

In many previous studies of parasitoids and other insects, cold anesthesia has been shown to cause STM disruption and resulting amnesia when applied and assessed shortly after learning (usually <1 h), with the behavioral expression of memory then returning as it is consolidated into other forms (ARM, LTM) (e.g., Xia, Feng & Guo, 1999; Van den Berg et al., 2011; Kruidhof et al., 2012); this is termed “retrograde amnesia”. Retrograde amnesia caused by anesthesia has not previously been tested in *T. basalis*. Thus, to maximize the probability of causing and detecting retrograde amnesia, we administered a treatment that would have caused memory disruption in all previous studies: we cold-anesthetized parasitoids immediately after learning and then re-tested them at different intervals. Parasitoids were trained on a first patch of host footprints and re-collected into a vial. Similar to the method employed by Van den Berg et al. (2011), the tube containing the wasp was then placed on ice for 15 s (causing the wasp to fall to the bottom of the tube and become immobile for up to 20 s following a return to room temperature, indicating anesthesia). Wasps were then tested in a second arena to measure their residence time (*n* = 32), with a minimum interval between anesthesia and retesting (i.e., test interval) of 15 min, and a maximum test interval of 5 h (mean ± SD  = 2.07 ± 1.45 h). We expected to see evidence of memory disruption when wasps were re-tested shortly after anesthesia (during the anesthesia-sensitive memory phase) and a decreasing effect with increasing time after anesthesia administration (i.e., as anesthesia-resistant memory phases were consolidated). As controls, we also tested wasps in parallel that had been cold-anesthetized at different intervals but were naïve with respect to host footprints (untrained) (*n* = 35), as well as both trained (*n* = 35) and untrained (*n* = 35) wasps that had not been anesthetized.

Previous studies in other parasitoids have succeeded in pharmaceutically disrupting memory derived from associative learning using the ATPase inhibitor ethacrynic acid (Schurmann et al., 2009) and the protein synthesis inhibitor anisomycin (Smid et al., 2007; Van den Berg et al., 2011; Kruidhof et al., 2012; Hoedjes & Smid, 2014) to block ARM and LTM, respectively. Procedures and product concentrations used were based on those used in these previous studies (Ethacrynic acid—Schurmann et al., 2009; Anisomycin—Hoedjes & Smid, 2014). Ethacrynic acid and anisomycin (Sigma Aldrich Italy) were diluted in sucrose/honey/water solution (ratio 0.1:1:1) to final concentrations of 5.0 mM and 1.0 mM, respectively. At least 1 h (maximum 3 h) before training, parasitoids housed in vials that had been deprived of food and water for 24 h were either offered solutions containing the chemical product (anisomycin or ethacrynic acid—“exposed”) or not (i.e., only sucrose/honey/water—“control”). As an improvement on some past studies, product consumption was verified (feeding typically lasted 15–30 s). Nearly all wasps (>90%) were observed to feed on the solution almost immediately; those that did not were not used. Exposed and control wasps were then trained on a first patch of host cues as described above, and then tested on a second patch over a continuous timeframe of 1-5 h (anisomycin mean ± SD  =2.56 ± 0.46_; ethacrynic acid =1.71 ± 0.18) later (test interval). These trained wasps (ethacrynic acid: *n* = 26 exposed, 26 control; anisomycin: *n* = 26 exposed, 23 control) were tested in parallel with untrained wasps that were naïve to host cues (ethacrynic acid: *n* = 26 exposed, 26 control; anisomycin: *n* = 27 exposed, 25 control) and their residence times were measured. Assignment of individuals to test intervals and trained/untrained treatments was randomized with respect to time since feeding on the solution.

### Statistical analyses

For all experiments, residence time data were analyzed with Cox proportional hazards models (hereafter “Cox models”), either standard or with mixed effects. These non-parametric survival models are well suited to time-to-event data (Crawley, 2012), which are seldom normally distributed and cannot be made to fit the assumptions of parametric models with transformations. Iterative likelihood ratio tests on full models were used to determine significance of each independent variable (main effects and interaction terms) (Crawley, 2012). The proportional hazards assumptions of the models were verified, and final model fit was assessed with residual plots.

First, using a Cox mixed effects model, we tested the dependence of *T. basalis* residence time during the four consecutive training sessions on training interval (the time spacing between training sessions), training number (first through fourth), and their interaction, including the individual wasp ID as a random effect. Since the interaction effect of training interval and training number was significant, we then split up the global model into four sub-models (one for each training number) to test the significance of training interval for each test interval. Next, we used Cox mixed effects models including individual wasp ID as a random effect to test whether residence times of each wasp, depending on training and testing intervals, increased during testing (relative to the final training session). Finally, using contrast tests within a Cox model, we tested whether the residence times of wasps in each training interval group was significantly different than that of untrained wasps trained in parallel (by including “untrained” as a fourth level of the training interval treatment and setting it as the intercept in the model). Here, we also tested for a potential interaction effect between training interval treatment and test interval to examine whether the differences in residence times between trained and untrained wasps depended on test interval.

For the three memory disruption experiments, standard Cox models were used to assess the dependence of residence time on treatment (exposed or not to memory blocking treatment), experience (trained or untrained), test interval (the interval between the end of the experience on the first patch and testing on the second patch) and the interaction of these factors.

All analyses were conducted with R version 3.1.3 (R Core Team, 2015).

## Results

### Tests for characteristics of habituation

**Figure 1:**
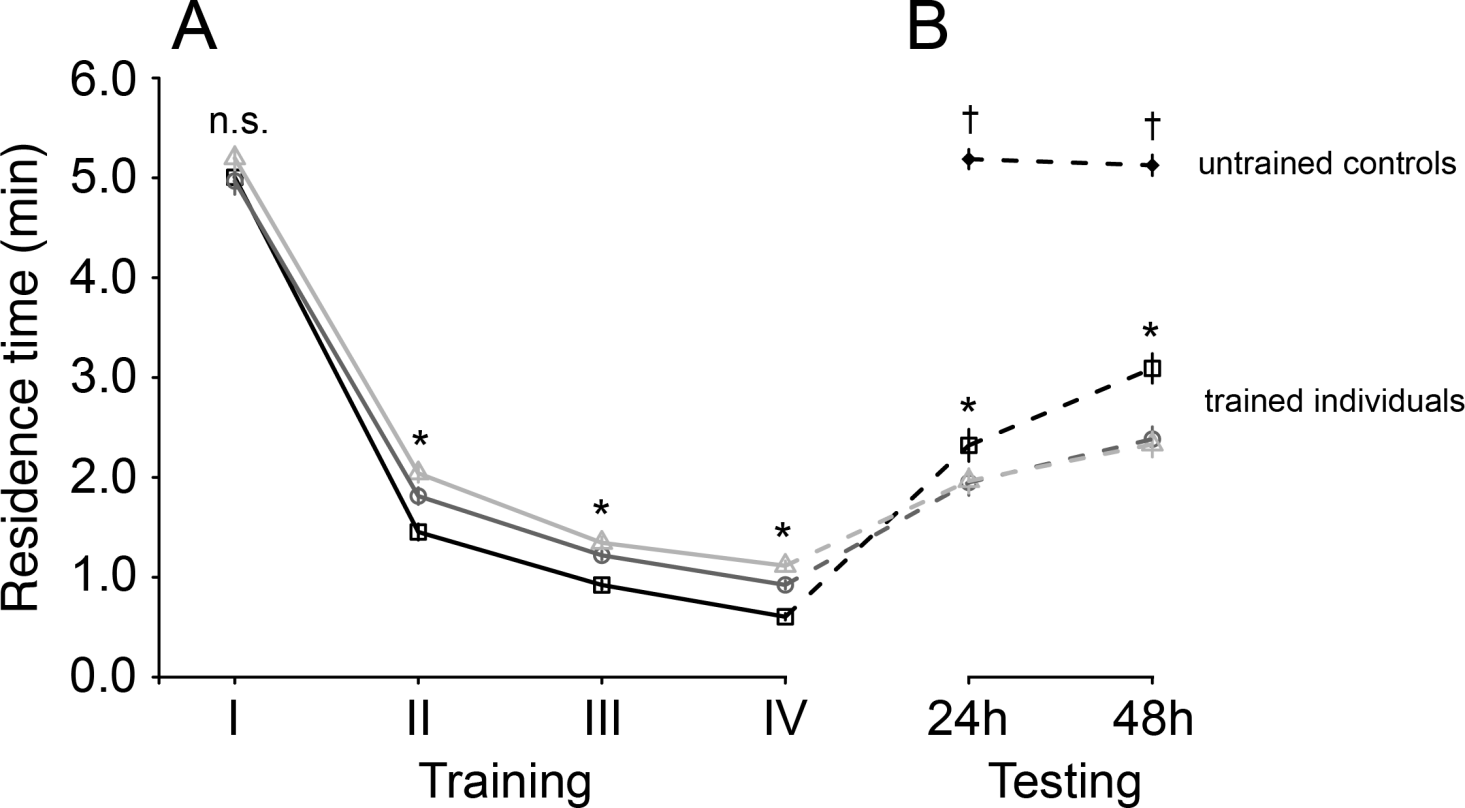
Mean residence time (±SE) of *Trissolcus basalis* on patches of *Nezara viridula* chemical footprints (A) over four consecutive training sessions and (B) when subsequently tested to measure spontaneous recovery 24 h or 48 h later and compared to untrained controls. Trained individuals were exposed to host footprints at one of three different training intervals: 60 min—light grey lines, empty triangles, 30 min—dark grey lines, empty circles, 15 min—black lines, empty squares. In (A) and (B), asterisks (*) (*p* < 0.0001) and “n.s.” (not significant) denote significance of comparisons among test interval treatments for a giving training or testing session (likelihood ratio tests on Cox models; n.s.—not significant; *— *p* < 0.0001). In (B), † denotes significant differences (*p* < 0.0001) between untrained individuals and trained individuals (all training intervals) at a given testing interval.

There was a significant interaction between the number of training sessions and test interval on the residence time of *T. basalis* (Cox mixed effects model, *χ*^2^ = 61.11, *df* = 6, *p* < 0.001). Residence times declined with each subsequent training session for all three training interval treatments, but to different degrees: residence times were similar among training interval treatments for the first training session, and were then significantly lower for shorter training intervals during the second through fourth training sessions (Fig. 1A).

After training, when wasps were tested, their residence times globally increased compared to those measured during the final training session (Cox mixed effects model, *χ*^2^ = 276.56, *df* = 4, *p* < 0.0001) (Fig. 1B). The magnitude of this increase depended on an interaction with both testing interval (*χ*^2^ = 9.08, *df* = 1, *p* = 0.0026) and training interval (*χ*^2^ = 74.60, *df* = 2, *p* < 0.0001): residence times recovered to a greater degree for wasps trained at shorter intervals, and had recovered more after 48 h than after 24 h (Fig. 1B).

Residence times of untrained wasps were overall higher than trained wasps (Cox model contrasts; all *p*-values <  0.0001), and were more similar to those tested 48 h after the last training session compared to those tested after 24 h (*χ*^2^ = 12.99, *df* = 3, *p* = 0.0047) (Fig. 1B).

### Memory disruption tests

**Figure 2:**
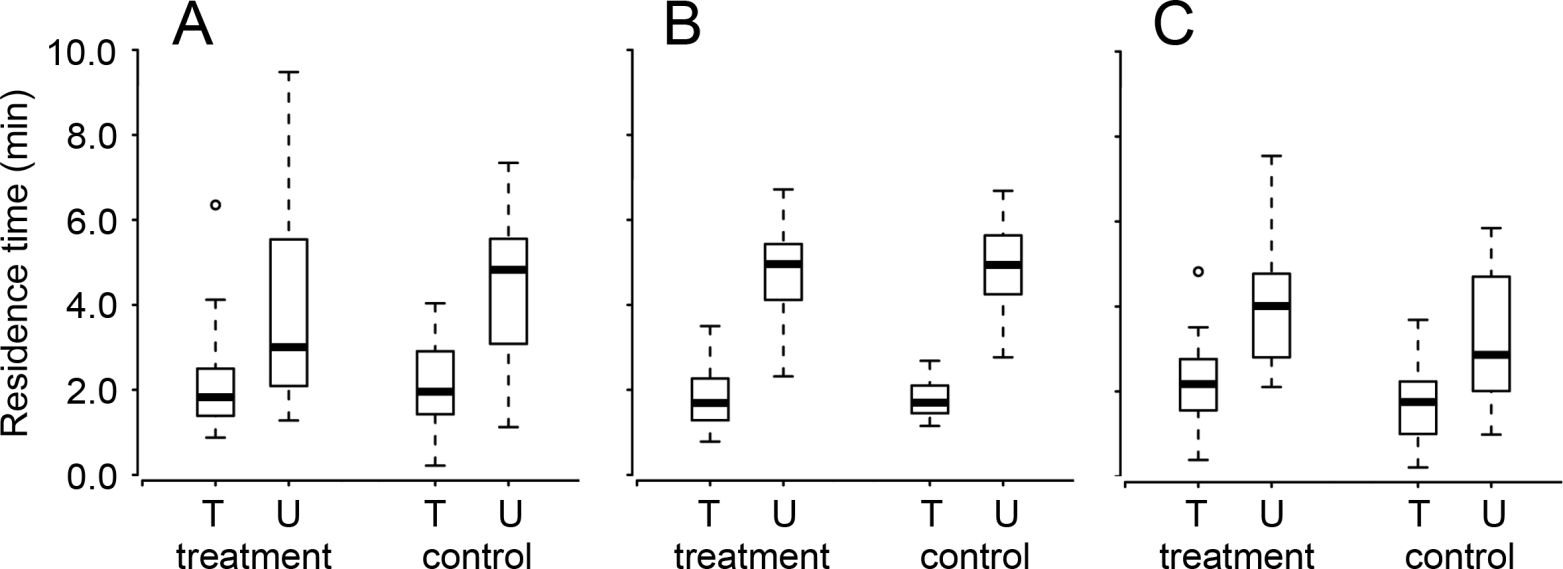
The residence times of once-trained (T) and untrained (U) *Trissolcus basalis* on patches of *Nezara viridula* chemical footprints when exposed (treatment) or not (control) to (A) cold anesthesia directly after training, or (B) ethacrynic acid or (C) anisomycin before training (or before testing for untrained wasps). Bolded horizontal lines show medians, boxes contain the 25th–50th percentiles, whiskers show the upper and lower quartiles, and points show outliers (more than 1.5 times the upper quartile). Results are pooled with respect to test interval. See Table 1 for statistical information.

When administered directly after a single training session, there was no effect of cold anesthesia or its interaction with training experience on the residence time of *T. basalis* (Table 1). Whether cold-anesthetized or not, trained wasps stayed on the patch for a significantly shorter time than untrained wasps (Table 1 and Fig. 2). The lack of effect of cold anesthesia on memory did not depend on the interval between anesthesia and testing: there was no effect of test interval or its interaction with any of the other factors (Table 1).

**Table 1:** Results of likelihood ratio tests on Cox models, showing the effects of treatment (Trt), experience (Exp), test interval (Ti), and their interactions on the residence time of *Trissolcus basalis* on patches of *Nezara viridula* chemical footprints, in three experiments to test the effects of cold anaesthesia, ethacrynic acid, and anisomycin on memory retention.

Experiment	Factor	*χ*^2^	*df*	*P*
Cold anesthesia	Trt	0.058	1	0.81
	Exp	46.58	1	<0.0001
	Ti	2.81	1	0.094
	Trt × Exp	0.27	1	0.60
	Trt × Ti	1.24	1	0.26
	Ti × Exp	0.99	1	0.32
	Trt × Ti × Exp	0.001	1	0.98
Ethacrynic acid	Trt	0.429	1	0.51
	Exp	118.152	1	<0.0001
	Ti	0.155	1	0.69
	Trt × Exp	1.09	1	0.30
	Trt ×Ti	0.51	1	0.48
	Ti ×Exp	0.25	1	0.62
	Trt ×Ti ×Exp	0.13	1	0.72
Anisomycin	Trt	4.43	1	0.035
	Exp	35.08	1	<0.0001
	Ti	0.039	1	0.84
	Trt × Exp	0.17	1	0.68
	Trt × Ti	0.821	1	0.36
	Ti ×Exp	0.066	1	0.79
	Trt × Ti × Exp	0.92	1	0.34

Ethacrynic acid ingestion prior to training had no effect on the subsequent residence time of *T. basalis* (Table 1 and Fig. 2). Trained wasps had a lower residence time on patches of host footprints (compared to untrained wasps) regardless of whether or not they had ingested ethacrynic acid, or the interval between training and testing (Table 1 and Fig. 2).

Anisomycin ingestion prior to training slightly increased the subsequent residence times (relative to controls) of both trained and untrained *T. basalis*, however this was the case for both trained and untrained wasps (untrained wasps having consistently longer residence times) (Table 1 and Fig. 2). As in the other two experiments, there was no effect of test interval or its interaction with any of the other factors (Table 1).

## Discussion

In order to advance in our understanding of optimal foraging strategies, it is important not only to test theoretical predictions, but also to examine underlying mechanistic assumptions. In testing what we term the “habituation assumption” underlying a widely accepted parasitoid foraging theory, we demonstrated that the foraging response of the parasitoid wasp *T. basalis* to host-derived contact kairomones (chemical footprints) did show some characteristics of habituation. These included both a declining responsiveness and a response recovery that was dependent on training interval. However, this putative habituation learning could not be disrupted by treatments that have been found to cause amnesia following associative learning in other species of parasitoids. Our study represents the first attempt to explicitly test for multiple characteristics of habituation in parasitoid foraging behavior.

If *T. basalis*’ behavioral response to indirect host-related cues constitutes habituation, residence time on patches of host footprints should (i) decline progressively with subsequent training sessions, and (ii) decline faster when training is administered at a shorter intervals (Thompson & Spencer, 1966; Rankin et al., 2009). This is exactly what we observed when we tested *T. basalis* on four consecutive patches of host cues. The intensity of the arrestment response of all groups of wasps declined asymptotically over the course of training, and in training interval-dependent manner (faster and more pronounced declines at higher training frequencies). While previous studies of parasitoids have shown progressive declines in responsiveness to host kairomones (both direct and indirect host-related cues) with repeated training (e.g., Waage, 1978; Bouchard & Cloutier, 1984; Strand & Vinson, 1982; Takabayashi & Takahashi, 1989; Peri et al., 2006), our study additionally demonstrates that responsiveness exhibits a training-interval dependent decline.

Spontaneous recovery of habituated behavioral responses and a positive dependence of the degree of spontaneous recovery on training interval are critical characteristics needed to distinguish habituation from sensory or motor fatigue (reviewed in Rankin et al., 2009).

We observed that the *T. basalis’* arrestment response partially recovered over time (increased in intensity, thus becoming more similar to that of untrained wasps). Furthermore, as expected, the degree of recovery was also positively related to the intervals separating prior training. There was little difference between the recovery observed for the two longer training intervals (compared to the two shortest training intervals), consistent with prior literature from mammalian and gastropod systems (Davis, 1970; Byrne, 1982) showing that frequency-dependent spontaneous recovery typically follows a pattern of diminishing returns with increasing training interval (i.e., the degree of recovery appears to reach an asymptote). In parasitoids, previous investigations have shown that decreased responsiveness to host kairomones can subsequently recover over time (e.g., Peri et al., 2006), but the degree of recovery has not been found to depend on training interval. This is yet another line of evidence suggesting that the learned response of *T. basalis* to contact kairomones of their hosts could constitute habituation.

Attempting to disrupt memory phases resulting from learning can give clues about potential underlying mechanisms (Hoedjes et al., 2011) and provide information about the environmental sensitivity of memory forms that are important for animal ecology (Abram et al., 2015). While no prior studies have attempted to disrupt habituation-derived memory in parasitoids, several studies have employed techniques such as cold anesthesia and pharmaceutical treatments to disrupt associative learning (e.g., Xia, Feng & Guo, 1998; Van den Berg et al., 2011), which may have similar underlying mechanisms to habituation (Duerr & Quinn, 1982; Engel & Wu, 1996; Cho, Heberlein & Wolf, 2004; Asztalos, Arora & Tully, 2007). Our attempts to disrupt *T. basalis* habituation with cold anesthesia, ATPase inhibition, and protein synthesis inhibition were all unsuccessful. Anisomycin caused wasps to stay on the patches of host footprints slightly longer overall, but this was not an effect on memory, *per se*, since the effect was of similar magnitude in both trained and untrained wasps. Some studies in other invertebrates (crabs, gastropods) have found that habituation learning can be blocked by protein synthesis inhibition (Pedreira, Dimant & Maldonado, 1996; Ezzeddine & Glanzman, 2003), while others have found that memory loss due to this inhibition may depend on the training context (Hermitte et al., 1999). Our results seem to indicate that habituation memory in *T. basalis*, at least with the training regime we used, cannot be blocked by protein synthesis inhibition, ATPase inactivation, or cold anesthesia. However, it is also possible that the timing of the treatments or the concentrations of the chemical agents we used were not optimal. For example, we would not observe retrograde amnesia if anesthesia-sensitive memory phases are consolidated into more stable phases within 15 min, the minimum time between training and testing in our bioassays. Past studies on other species of parasitoids (as well as other insects) have shown that anesthesia-sensitive memory persists for at least one and up to two hours (e.g., Xia, Feng & Guo, 1999; Van Alphen, Bernstein & Driessen, 2003; Kruidhof et al., 2012; Schurmann, Kugel & Steidle, 2015), but we cannot exclude the possibility that it could be much shorter in *T. basalis*. For the pharmaceutical treatments, we based the concentrations of our chemical treatments on past experiments in other parasitoid species (Schurmann et al., 2009; Hoedjes & Smid, 2014), but we acknowledge the difficulty in assessing whether the concentrations we employed in our specific study system were ideal until further studies are conducted.

While we demonstrated four critical characteristics of habituation in the foraging behavior of parasitoid, there are still several remaining characteristics that we did not test, including stimulus generalization (i.e., the specificity of the stimulus in causing the habituated response) and dishabituation (i.e., a recovery of the habituated response following presentation of a different stimulus) (see Rankin et al., 2009). Future studies should investigate whether animal foraging behaviors show these additional characteristics of habituation, and whether this putative habituation can be distinguished from a negative associative learning process (absence of suitable hosts as an unconditioned stimulus), which was not addressed in our study. It will also be important to introduce more ecological complexity and realism by examining characteristics of habituation in the context of parasitoids exploiting patches containing both kairomones and hosts, and over longer timescales.

##  Supplemental Information

10.7717/peerj.3097/supp-1 Data S1 Raw experimental data

## Additional Information and Declarations

### Data Availability

The following information was supplied regarding data availability: The raw data has been supplied as Data S1.
